# Cefiderocol Resistance Conferred by Plasmid-Located Ferric Citrate Transport System in KPC-Producing *Klebsiella pneumoniae*

**DOI:** 10.3201/eid3101.241426

**Published:** 2025-01

**Authors:** Riccardo Polani, Alice De Francesco, Dario Tomolillo, Irene Artuso, Michele Equestre, Rita Trirocco, Gabriele Arcari, Guido Antonelli, Laura Villa, Gianni Prosseda, Paolo Visca, Alessandra Carattoli

**Affiliations:** Sapienza University of Rome, Rome, Italy (R. Polani, A. De Francesco, D. Tomolillo, R. Trirocco, G. Arcari, G. Antonelli, G. Prosseda, A. Carattoli); University of Pavia, Pavia, ltaly (D. Tomolillo); Istituto Superiore di Sanità, Rome (I. Artuso, M. Equestre, L. Villa); University of Insubria, Varese, Italy (G. Arcari); Sapienza University Hospital “Policlinico Umberto I,” Rome (G. Antonelli); Roma Tre University, Rome (P. Visca)

**Keywords:** Klebsiella pneumoniae, carbapenemase-producing Klebsiella pneumoniae, KPC, cefiderocol, antimicrobial resistance, siderophore, ferric citrate-dependent iron transport system, bacteria, Italy

## Abstract

Cefiderocol (FDC), a siderophore-cephalosporin conjugate, is the newest option for treating infection with carbapenem-resistant gram-negative bacteria. We identified a novel mechanism contributing to decreased FDC susceptibility in *Klebsiella pneumoniae* clinical isolates. The mechanism involves 2 coresident plasmids: pKpQIL, carrying variants of *bla*_KPC_ carbapenemase gene, and pKPN, carrying the ferric citrate transport (FEC) system. We observed increasing FDC MICs in an *Escherichia coli* model system carrying different natural pKpQIL plasmids, encoding different *K. pneumoniae* carbapenemase (KPC) variants, in combination with a conjugative low copy number vector carrying the *fec* gene cluster from pKPN. We observed transcriptional repression of *fiu*, *cirA*, *fepA*, and *fhuA* siderophore receptor genes in *bla*_KPC_–*fec*–*E. coli* cells treated with ferric citrate. Screening of 27,793 *K. pneumoniae* whole-genome sequences revealed that the *fec* cluster occurs frequently in some globally distributed different KPC-producing *K. pneumoniae* clones (sequence types 258, 14, 45, and 512), contributing to reduced FDC susceptibility.

*Klebsiella pneumoniae* is 1 of 6 global leading ESKAPE pathogens (*Enterococcus faecium*, *Staphylococcus aureus*, *K. pneumoniae*, *Acinetobacter baumannii*, *Pseudomonas aeruginosa*, and *Enterobacter* spp.) associated with high morbidity and mortality rates and antimicrobial resistance (*1*). Among those pathogens, the World Health Organization designated carbapenem-resistant *K. pneumoniae* as a priority pathogen (*2*). In 1996, *K. pneumoniae* carbapenemase (KPC) was identified in *K. pneumoniae* sequence type (ST) 258 in the United States and then spread worldwide (*3*–*5*). To date, *bla*_KPC_ gene variants have been found on different plasmid types; pKpQIL is prevalent in successful clones ST258 and ST512 (*6*,*7*).

Since 2015, ceftazidime/avibactam (CZA) has been available for treatment of complicated and deep-seated infections, including bacteremia caused by carbapenemase-producing Enterobacterales in adults (*8*). CZA combines ceftazidime, a third-generation cephalosporin, and avibactam, a β-lactamase inhibitor (*9*). Since 2018, KPC-producing CZA-resistant *K. pneumoniae* strains have been described (*10*,*11*) carrying mutations in the Ω-loop of the KPC protein, such as KPC-31 (*12*,*13*).

Cefiderocol (FDC), approved by the US Food and Drug Administration in 2020, is available to treat Enterobacterales, *Acinetobacter baumannii*, and *Pseudomonas aeruginosa* invasive infections caused by carbapenem- and CZA-resistant strains (https://www.accessdata.fda.gov/drugsatfda_docs/nda/2020/209445Orig1s002.pdf). FDC is a cephalosporin linked with a chlorocatechol group, which provides the drug with a siderophore-like moiety that serves as a Trojan horse to gain access to the bacterial periplasm. The chlorocatechol group is thought to enhance the entry of FDC in the bacterial cell through energy-dependent uptake by chromosome-encoded ferric siderophore transporters (*14*,*15*). To determine the mechanism of FDC resistance in KPC-producing *K. pneumoniae*, we analyzed the contribution of a plasmid-encoded ferric citrate uptake system (FEC), which acts synergistically with CZA-resistant KPC variants.

## Materials and Methods

### *K. pneumoniae* Isolates

*K. pneumoniae* strains PL1, PL2, PL3, and PL4 were isolated at the University Hospital Policlinico Umberto I, Rome, Italy, from blood samples of 1 patient during 1 month of hospitalization. The strains were processed for routine diagnostics and compared with 31 previously described strains (Appendix 1, Table 1).

### FDC Antimicrobial Susceptibility

We determined FDC MICs by using ComASP (Liofilchem, https://www.liofilchem.com) or by using a ComASP panel enriching iron-depleted cation-adjusted Mueller-Hinton broth with 0.5 μM or 5.0 µM ammonium ferric citrate or trisodium citrate dihydrate (Merck KGaA, https://www.emdgroup.com). We preliminarily determined the citrate concentration used for induction of the FEC system in the *E. coli* model, and 0.5 µM ammonium ferric citrate was the minimal concentration that did not increase the FDC MIC by >1-fold in treated compared with untreated *E. coli* strains.

### Whole-Genome Sequencing

We purified genomic DNA by using the MagaBio Bacterium DNA Purification Kit III (Hangzhou Bioer Technology Co., https://www.bioer.com) and GenePure Pro Nucleic Acid Purification System (Bioer Technology, https://www.bioer.com.cn), and we used NanoDrop One Microvolume UV-Vis Spectrophotometer and Qubit 4.0 Fluorometer (Invitrogen, https://www.thermofisher.com) to assess. We prepared DNA libraries by using the Nextera XT DNA Library Preparation Kit and loaded them onto a MiSeq Reagent Kit v.3 cartridge (Illumina, https://www.illumina.com). We performed paired-end sequencing on an Illumina MiSeq platform, with a read length of 2 × 300 bp. We trimmed resulting reads by using trimmomatic (*16*) and assembled them by using SPAdes (*17*).

### Long-Reads Sequencing

We performed Oxford Nanopore Technologies (ONT) sequencing on a MinION Mk1C sequencing platform (https://nanoporetech.com). We extracted genomic DNA by using a Monarch HMW DNA Extraction Kit for Tissue (NEB, https://www.neb.com) and prepared libraries by using ONT Rapid Barcoding Kit 24 and sequencing on R10.4.1 flow cells. We performed long-read assemblies by using Flye (*18*).

We analyzed hybrid assembly obtained by Unicycler (*19*) and Hybracter (*20*) by using Staramr (https://bio.tools/staramr). We annotated genomes by using Prokka (*21*) and identified single-nucleotide polymorphisms by using Snippy (https://github.com/tseemann/snippy).

### pKpQIL Plasmid Transformation

We introduced plasmid DNA extracted by using a Pureyield Plasmid Midiprep System (Promega, https://www.promega.com) in chemically competent *E. coli* Max efficiency DH5-α cells (Thermo Fisher Scientiﬁc, https://www.thermofisher.com). We selected transformants on Luria broth (LB) agar plates containing ceftazidime (6 mg/L).

### R69c and R69c-FEC Plasmid Assembly

We obtained the R69c vector (GenBank accession no. PQ130559) by cloning the chloramphenicol resistance *catA* gene amplified from Addgene plasmid #46569 in the *Sma*I site of R69#1 (European patent EP3541942B1, A. Carattoli, A. Endimiani, https://patents.google.com/patent/EP3541942B1/de?oq=EP3541942B1, accessed November 29, 2024). We obtained PCR products by using PCRBIO VeriFi Polymerase (PCR Biosystems, https://pcrbio.com) and primers listed in Appendix 1, Table 2.

**Figure 1:**
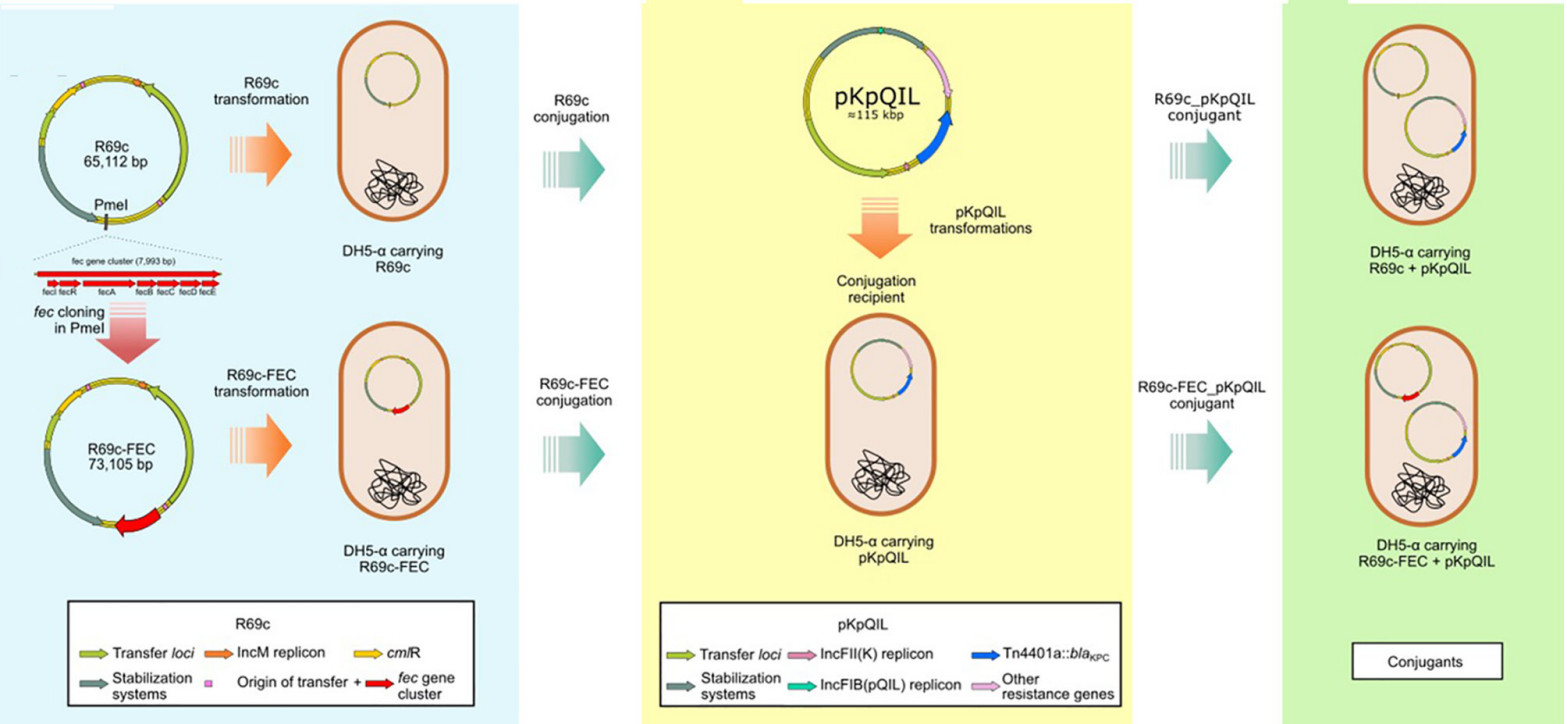
Schematic representation of R69c and R69c-FEC conjugation in *Escherichia coli* DH5-α recipient carrying the pKpQIL plasmids R69c and R69c-FEC and pKpQIL major features in study of cefiderocol resistance conferred by plasmid-located ferric citrate transport system in KPC-producing *Klebsiella pneumoniae*. The left panel (blue) represents construction of the R69c and R69c-FEC donor vectors, both introduced by transformation in *E. coli* DH5-α chemically competent cells. The central panel (yellow) shows the pKpQIL transformation of *E. coli* DH5-α chemically competent cells with different pKpQIL natural plasmids extracted from *K. pneumoniae* strains. The right panel (green) represents the exconjugant pairs obtained by conjugation of the R69c vectors into the recipients carrying the different pKpQIL plasmids. FEC, ferric citrate transport system. KPC, *Klebsiella pneumoniae *carbapenemase*.*

We obtained the R69c-FEC vector (GenBank accession no. PQ085644) by cloning the 7,993-bp *fec* PCR product (Appendix 1, Figure 1) from the PL3 strain and cloned it in R69c PmeI site. Both clonings were performed by using GeneArt Gibson Assembly HiFi Master Mix (Thermo Fisher Scientific).

We extracted plasmids by using ZymoPURE II Plasmid Maxiprep Kit (Zymo Research, https://www.zymoresearch.com) concentrated by Microcon DNA Fast Flow device (Merck KGaA). We performed transformations by using MAX Efficiency DH5α-T1R Competent Cells (Thermo Fisher Scientific) selected on chloramphenicol 25 mg/L LB agar plates.

### Plasmid Conjugation

We grew donor and recipient strains separately in LB broth without antibiotics at an optical density of 1 McFarland. We pooled 50 μL of each culture and dropped 20 μL of the mixture on an LB plate without antibiotics and incubated it at 37°C for 6–10 hours. A patina from the conjugation spot was diluted and plated on LB agar plates containing 25 mg/L chloramphenicol and 6 mg/L CAZ. We sequenced selected positive exconjugants by using Illumina and ONT procedures.

### RNA Isolation and Quantitative Reverse Transcription PCR

We conducted bacterial RNA purification on R69c/R69c-FEC-PL3 exconjugant pairs grown in liquid iron-depleted media in the presence of 0 μM, 0.5 μM, or 5.0 μM ammonium ferric citrate by using a hot phenol extraction method (*22*). We conducted cDNA synthesis and quantitative reverse transcription PCR analyses on a 7300 real-time PCR system (Applied Biosystems, https://www.thermofisher.com) (*23*). We obtained cDNA for *nusA* (used as normalizer), *fiu*, *cirA*, *fepA*, *fhuA*, or *fecA* genes (Appendix 1, Table 2).

### Global Distribution of *fec* Gene Cluster

As of July 4, 2024, we downloaded 27,993 *K. pneumoniae* genomes from the Pasteur Institute BIGSdb *Klebsiella pneumoniae* database (https://bigsdb.pasteur.fr) together with their metadata (Appendix 2, Table). We included 35 strains from our study and previous studies in the collection (Appendix 1, Table 1).

We screened *K. pneumoniae* genomes for the *fec* operon by using the BLASTN tool in the Bacterial Isolate Genome Sequence BIGSdb database (https://bigsdb.pasteur.fr). We used as reference the *fecABCDE* operon and *fecIR* regulatory genes from *K. pneumoniae* PL3 (GenBank accession no. CP168103, nt positions 113339–121331). The *fec* gene cluster was considered present if the E-value was <1e^−10^ and identity was >85% across >90% of the sequence length.

We assessed the presence of the FIB(K) replicon (GenBank accession no. JN233704) by using the BLASTN tool. We also determined the presence of *bla*_VIM_, *bla*_NDM_, and *bla*_KPC_ genes by using the gene presence tool at the Pasteur Institute website (https://bigsdb.pasteur.fr) with minimum percentage identity of 95% and a minimum percentage alignment of 99%.

### Phylogenetic Analysis

We used the Prokka tool (*20*) to annotate 2,493 genomes belonging to ST101, ST307, and ST512 (Appendix 3, Table). We used Roary (*24*) to generate core-genome alignments, using MAFFT (*25*), accordingly, to the ST and IQ-TREE 2 (*26*) to construct phylogenetic trees with 1,000 ultrafast bootstrap iterations.

## Results

### Effect of KPC Variants on Reduced Susceptibility to FDC

**Figure 2:**
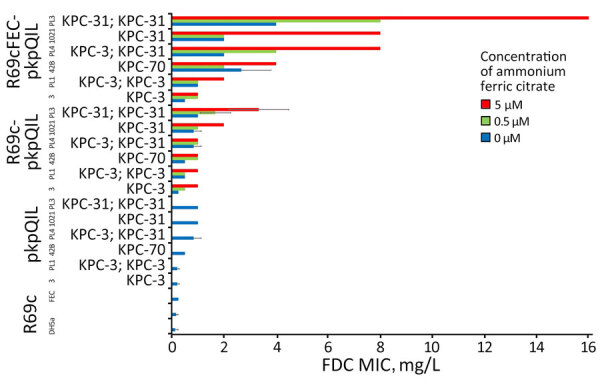
FDC MICs of KPC-producing *Escherichia coli *in a study of cefiderocol resistance conferred by plasmid-located ferric citrate transport system in KPC-producing *Klebsiella pneumoniae*. FDC susceptibility tests were performed according to manufacturer directives, with concentrations of 0 µM, 0.5 µM, and 5 µM ammonium ferric citrate on *Escherichia coli *DH5-α cells carrying different combinations of pKpQIL, R69c, and R69c-FEC plasmids. FDC, cefiderocol; KPC, *Klebsiella pneumoniae *carbapenemase.

**Figure 3:**
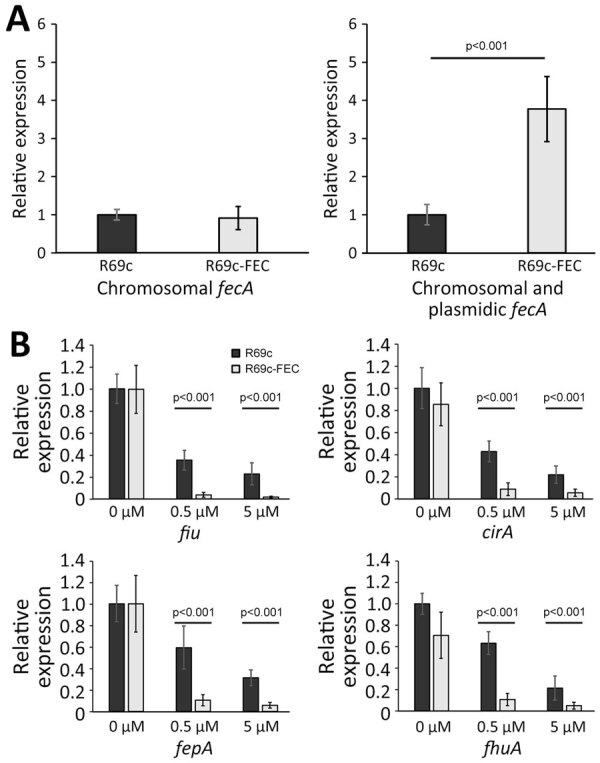
Expression analysis of siderophore receptor genes in the presence and absence of plasmidic *fec *gene cluster and ferric citric inducer in an *Escherichia coli *model used in a study of cefiderocol resistance conferred by plasmid-located ferric citrate transport system KPC-producing *Klebsiella pneumoniae. *A) Transcription of the *fecA *genes in the DH5-α strain carrying R69c or R69c- FEC, determined by using primer pairs able to discern the chromosomal *fecA *allele from the *K. pneumoniae fecA *gene in the *fecABCDE *operon or a primer pair recognizing both chromosomal and plasmidic *fecA *alleles (Appendix 1 Table 2). The relative quantitative analysis of the transcripts was based on the 2^−ΔΔCT^ method (*31*). In both bar graphs, the relative values were calculated with respect to the transcript level observed in the R69c carrying strains and set to 1. B) Transcription of the siderophore receptor genes *fiu*, *cirA*, *fepA*, and *fhuA *in the R69c-FEC and R69c strains grown in the absence of ferric citrate or in the presence of 0.5 μM or 5.0 μM ferric citrate, relative to the R69c strain grown without ferric citrate, which is set to 1. The relative quantitative analysis of the transcripts was based on the 2^−ΔΔCT^ method (*31*). Error bars represent SDs. Statistical significance was determined by using a paired 2-tailed Student *t*-test comparing the dataset obtained from the 2 strains grown under the same conditions. FEC, ferric citrate transport system; KPC, *Klebsiella pneumoniae *carbapenemase.

Our initial aim with this study was to explain the different levels of FDC resistance in 4 ST512 *K. pneumoniae* clinical isolates (PL1, PL2, PL3, and PL4) from 1 patient during 1 month of hospitalization. PL3 was resistant to FDC (MIC 4 mg/L), whereas FDC MICs PL1, PL2, and PL4 were below the breakpoint value (0.5–2.0 mg/L; Appendix 1, Table 1). The PL1–4 genomes were closely related at the chromosomal level (6–22 single-nucleotide polymorphisms and indels on the chromosome; Appendix 4, Table) but showed different *bla*_KPC_ genes and plasmid content. All PL1–4 *K. pneumoniae* strains carried different pKpQIL variants plus an IncX3 plasmid and a small ColRNAI plasmid. The pKpQIL-PL1 plasmid harbored 2 copies of the *bla*_KPC-3_ gene, both pKpQIL-PL2 and pKpQIL-PL4 carried 1 copy of *bla*_KPC-3_ and 1 copy of *bla*_KPC-31_, whereas pKpQIL-PL3 carried 2 copies of *bla*_KPC-31_ (Appendix 1, Figure 2). Isolates PL2 and PL3 were also enriched with the pKPN plasmid, which was absent in PL1 and PL4. In PL2, pKPN was fused with pKpQIL-PL2 in the *tnpR-*FIIK_1_ integration site, forming a 263,486-bp plasmid. The hybrid pKPN-pKpQIL-PL2 plasmid was not transferable by transformation or conjugation and could not be further studied. In PL3, the stand-alone pKPN plasmid had acquired the *fec* gene cluster encoding for a FEC system. The *fec* gene cluster was unique to the pKPN plasmid in PL3 and was absent in PL1, PL2, and PL4. The *fec* genes mapped (alongside an ABC glutathione transporter, the *lacZ*, *lacY*, and *lacI* genes) between 2 IS*4321* elements positioned between the *tnpR* gene and the FIIK replicon (Appendix 1, Figure 3).

Acquisition of *fec* genes was suspected to correlate with increased FDC MICs of PL3. We then measured FDC MICs for all KPC-producing *K. pneumoniae* clinical strains in our collection isolated since 2018 with a completely sequenced genome, in search for *fec*, other siderophore receptors, and porin gene sequences in their genomes (Appendix 1, Table 1).

FDC MICs were 0.25–32 mg/L. The lowest (0.25 mg/L) was measured in a strain producing KPC-3 (strain 3), encoding the yersiniabactin siderophore-dependent iron uptake system, the wild-type CirA and Fiu siderophore receptors, and a wild-type OmpK36 porin (*27*). The highest FDC MIC (32 mg/L) was for a strain producing VIM (Verona integron-encoded metallo-β-lactamase) and KPC carbapenemases (strain 0296), characterized by a nonsense mutation in the gene encoding the siderophore receptor CirA (E133X) (*28*).

Presence of CZA-resistant variant KPC-31, KPC-70, or KPC-68 was associated with high MICs (4 mg/L). Higher MICs (1–2 mg/L) for FDC were obtained in *E. coli* Top-10 transformed with the *bla*_KPC-31_, *bla*_KPC-70_, or *bla*_KPC-68_ genes, respectively cloned in the pTopo-KanR vector (Appendix 1, Table 1).

In addition to the role of KPC variants in determining FDC resistance levels, we noticed that *K. pneumoniae* exhibiting higher FDC MICs were positive for the FEC system (*29*), carried by the pKPN plasmid (*30*). The *fec* gene cluster was identified in 13/35 isolates from the collection. Eleven KPC-31–producing strains belonging to different STs showed FDC MICs of 1–2 mg/L, but 2 KPC-31 strains carrying the *fec* gene cluster reached a MIC of 4.0 mg/L (Appendix 1, Table 1). We hypothesized that the plasmid-borne FEC system could reduce the susceptibility to FDC in *K. pneumoniae* clinical isolates.

### *E. coli* Model

We constructed an in vitro model in isogenic *E. coli* DH5-α cells, suitable for studying the effect of the FEC transport system on FDC resistance levels, excluding the contribution of other resistance factors, siderophore receptors, and porins encoded by the *K. pneumoniae* clinical isolates. The model consisted of the 2-step introduction in *E. coli* DH5-α cell of pKpQIL plasmids carrying different KPC variants and an engineered 64-Kb R69c self-conjugative plasmid vector carrying the *K. pneumoniae fec* gene cluster.

First, selected pKpQIL plasmid variants were individually introduced by transformation into chemically competent *E. coli* DH5-α cells. We tested the model on pKpQIL transformants obtained from strains 3, 42B, and 1021, encoding the KPC-3, KPC-70, and KPC-31 variants, respectively (Appendix 1, Table 3). We also studied pKpQIL transformants carrying copies of the *bla*_KPC_ gene obtained from *K. pneumoniae* PL1, PL3, and PL4 strains.

**Figure 4:**
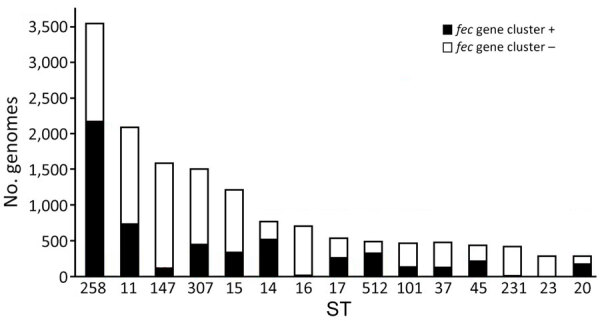
Distribution of the *fec* gene cluster among prevalent STs of *Klebsiella pneumoniae.* Distribution of the *fec* gene cluster, represented by a black bar (number of positive genomes) or a white bar (number of negative genomes), across the total analyzed genomes for prevalent STs in the *K. pneumoniae* database (Appendix 3, Table). ST, sequence type.

Second, the 64-Kb R69c, self-conjugative plasmid vector was engineered to host *fec* genes. The R69c vector is a derivative of the R69 IncM natural plasmid (GenBank accession no. KM406488) and carries the *catA* gene, conferring chloramphenicol resistance. R69c is a self-conjugative, low-copy-number plasmid that simulates the horizontal transmission of the pKPN natural plasmid. It carries all the genes enabling conjugation at high efficiency (1×10^−2^ conjugants/recipient cell), conferring stabilization, and the IncM replicon for replication and copy number control (Appendix 1, Figure 1). The plasmid enables cloning and transfer of genetic determinants at low copy numbers by conjugation. Because R69c contains a stabilization system, after chloramphenicol selection of transconjugants, the recipient *E. coli* clones do not need further antimicrobial selection to ensure plasmid maintenance. The *fecIR-fecABCDE* gene cluster, including the Fur and iron-regulated promoter regions (*32**,**33*) (Appendix 1, Figure 4), were amplifiedcfrom the pKPN-PL3 *K. pneumoniae *plasmid. The re- sulting PCR product of 7,993 bp, consisting of the *fecIR *promoter region, the *fecI *and *fecR *regulatory genes, and the *fecA*, *fecB*, *fecC*, *fecD*, and *fecE *genes encoding the complete FEC system with internal regulatory regions, was cloned in the unique PmeI restriction site of R69c, obtaining the 73 Kb vector, named R69c-FEC (Appendix 1, Figure 1).

### Evaluation of FDC MICs in the *E*. *coli* Model

We introduced R69c and R69c-FEC vectors by conjugation in DH5-α pKpQIL transformants (Appendix 1, Table 3**)** by obtaining pairs of exconjugants carrying the same pKpQIL variant and, alternatively, the R69c vector with or without the cloned *fec* gene cluster (Figure 1**)**. As an *E. coli* K-12 derivative, strain DH5-α cells possess the chromosomal *fec* gene cluster (94.51% nt identity, 96% coverage; Appendix 1, Figure 4). Higher FDC MICs were invariably observed for exconjugants carrying the R69c-FEC, relative to the isogenic strain carrying the same pKpQIL with R69c lacking the *fec* gene cluster (Figure 2). The highest FDC MIC of 4 mg/L in iron-depleted media was obtained for the exconjugant carrying both pKpQIL-PL3 and R69c-FEC. The respective comparative exconjugant carrying the R69c reached an FDC MIC of 1 mg/L.

We tested in vitro susceptibility to FDC of R69c and R69c-FEC exconjugant pairs under inducing conditions by adding ferric citrate, which serves as substrate and inducer of the FEC system. In our experimental conditions, 0.5 μM or 5.0 μM of ferric citrate increased the FDC MICs of R69c/R69c-FEC DH5-α cells relative to untreated cells. The effect in strains carrying the R69c vector without the cloned *fec* gene cluster was attributed to the chromosomal *fec* gene cluster in the DH5-α background. We did not observe increased FDC MICs when using 0 μM, 0.5 μM, and 5.0 μM trisodium citrate dihydrate without iron (data not shown). The highest FDC MICs were reached by R69c-FEC-PL3 (MICs 8 in the presence of 0.5 μM and 16 mg/L in the presence of 5.0 μM ferric citrate). Under the same conditions, R69c-PL3 FDC MICs were 2 and 4 mg/L (Figure 2; Appendix 1, Table 3). The experiments performed with R69c-FEC/R69c-1021, R69c-FEC/R69c-42B, and R69c-FEC/R69c-PL4 pairs (Appendix 1, Table 3) demonstrated that the presence of an inhibitor-resistant KPC variant, such as KPC-31 and KPC-70 in combination with the plasmid-located *fec* gene cluster was sufficient to reduce FDC susceptibility (MIC >2.0 mg/L) relative to the R69c controls lacking the *fec* gene cluster. Further FDC MIC increment can be obtained by treatment with ferric citrate (MIC≥4.0 mg/L).

Because the FEC system is implicated in iron delivery to the cell, we compared the mRNA transcription of *fiu*, *cirA*, *fepA*, and *fhuA* siderophore receptor genes and the endogenous *fec* gene cluster in DH5-α carrying the R69c-FEC plasmid with DH5-α carrying the R69c, both in the presence or absence of ferric citrate. The expression of the R69c-located *fecA* cluster was estimated to be 3-fold higher than that of the chromosomal DH5-α *fec* cluster (Figure 3, panel A).

We observed a substantial reduction of the expression of *fiu*, *cirA*, *fepA*, and *fhuA* siderophore receptor genes growing the cells in the presence of 0.5 μM and 5.0 μM ferric citrate (relative to no ferric citrate). The inhibition of *fiu*, *cirA*, *fepA*, and *fhuA* gene expression was almost complete (90%) in cells carrying the R69c-FEC plasmid and only partial in R69c-carrying cells (30%–40%, R69c-FEC vs. R69c; Figure 3, panel B). The markedly reduced expression of ferrisiderophore receptor genes caused by the cloned *K. pneumoniae fec* gene cluster correlated with the higher FDC MICs observed in R69c-FEC-positive strains in those conditions.

### Prevalence of FEC Siderophore Transport System in *K. pneumoniae*

We performed global screening of the *fec* gene cluster in 27,793 *K. pneumoniae* whole-genome sequences downloaded from the Pasteur Institute database (https://bigsdb.pasteur.fr/klebsiella; Appendix 2, Table). The resulting dataset included genomes of globally prevalent clones (Figure 4).

**Figure 5:**
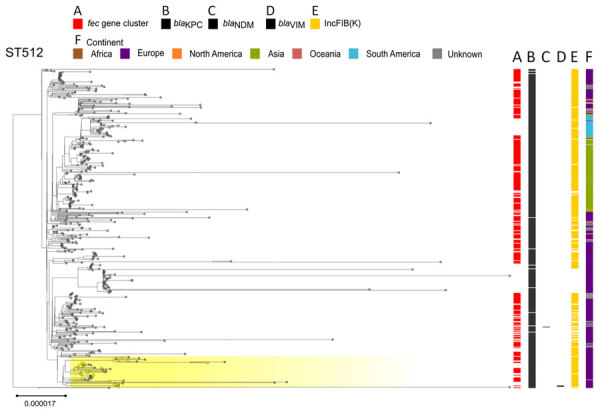
Phylogenetic analysis of *Klebsiella pneumoniae* ST512 based on core-genome alignment of 510 *K. pneumoniae* ST512 isolates. The tree is midpoint rooted, and the scale bar represents the number of substitutions per site. The presence of the *fec* operon is indicated in red; *bla*_KPC_, *bla*_VIM_, and *bla*_NDM_ genes in black; and the FIB(K) replicon in orange. Yellow shading indicates genomes sequenced in this study or our previous studies (Appendix 1, Table 1). The best-fit model was selected by ModelFinder (http://www.iqtree.org/ModelFinder), The tree was visualized with Microreact (https://microreact.org) and adjusted by using the InkScape software (https://www.inkscape.org). ST, sequence type.

**Figure 6:**
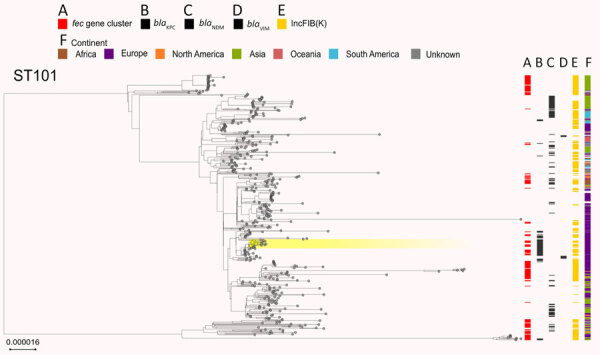
Phylogenetic analysis based on core-genome alignments of 468 *Klebsiella pneumoniae* ST101 isolates in study of cefiderocol resistance conferred by plasmid-located ferric citrate transport system in *K. pneumoniae* carbapenemase–producing *K. pneumoniae.* The trees are midpoint rooted, and the scale bar represents the number of substitutions per site. The presence of the *fec* operon is indicated in red; *bla*_KPC_, *bla*_VIM_, and *bla*_NDM_ genes in black; and the FIB(K) replicon in orange. Yellow shading indicates genomes sequenced in this study or our previous studies (Appendix 1, Table 1). The best-fit model was selected by ModelFinder (*34*). The trees were visualized with Microreact (https://microreact.org) and adjusted by using the InkScape software (https://www.inkscape.org). ST, sequence type.

**Figure 7:**
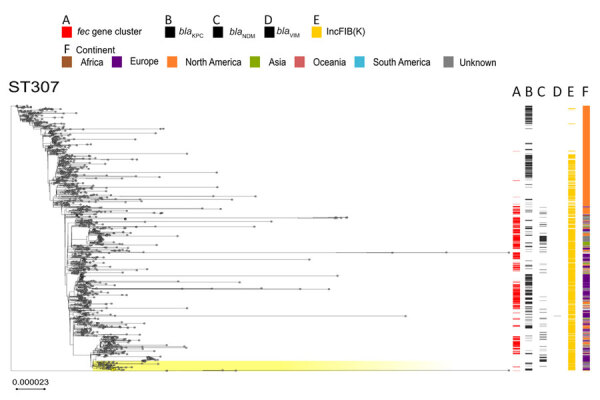
Phylogenetic analysis based on core-genome alignments of 1,516 *Klebsiella pneumoniae* ST307 isolates in a study of cefiderocol resistance conferred by plasmid-located ferric citrate transport system in *K. pneumoniae* carbapenemase–producing *K. pneumoniae.* The trees are midpoint rooted, and the scale bar represents the number of substitutions per site. The presence of the *fec* operon is indicated in red; *bla*_KPC_, *bla*_VIM_, and *bla*_NDM_ genes in black; and the FIB(K) replicon in orange. Yellow shading indicates genomes sequenced in this study or our previous studies (Appendix 1, Table 1). The best-fit model was selected by ModelFinder (*34*). The trees were visualized with Microreact (https://microreact.org) and adjusted by using the InkScape software (https://www.inkscape.org). ST, sequence type.

The *fec* gene cluster was detected in 10,672 isolates across the dataset (38.4%), of which 2,658 (24.0%) carried a *fec* cluster with 100% nt identity and 100% coverage compared with that of *K. pneumoniae* PL3 (Appendix 5, Table). The *fec* gene cluster was unevenly spread among the 15 most prevalent STs. The *fec* gene cluster was carried by a majority (>50%) of ST258, ST14, ST45, and ST512 isolates and a minority (<10%) of ST16, ST147, ST23, and ST231 isolates. The *fec* genes were more prevalent in ST512 than in the general *K. pneumoniae* population (68% of *fec-*carrying ST512 genomes vs. 37.87% of *fec-*carrying non-ST512 genomes; p<0.00001 by χ^2^ test). Most ST512 genomes carried 1 copy of the *bla*_KPC_ carbapenemase gene (Figure 5), whereas the co-presence of *fec* and *bla*_KPC_ was less frequent in other clones such as ST101 and ST307 (Figures 6, 7).

Our data suggest that the risk for developing resistance to FDC may be higher in clones like ST512, which more frequently carry the *fec* gene cluster along with the *bla*_KPC_ gene. The specific FIB(K) replicon, marking the pKPN plasmid, was detected in 16,325 (58.3%) genomes, of which 2,585 (15.8%) also carried the *fec* cluster of *K. pneumoniae* PL3 (Appendix 2, Table).

## Discussion

In vitro studies on FDC resistance have unveiled that CZA-resistant KPC variants (e.g., KPC-31) and class B metallo-β-lactamases have a role in FDC resistance (*35*). Furthermore, mutational inactivation of CirA and Fiu siderophore receptors has been demonstrated to reduce FDC susceptibility in vitro and in vivo (*362*,*37*).

With this study, our first hypothesis was to attribute FDC resistance of PL3 to *bla*_KPC-31_ gene duplication (*38*–*40*). Subsequently, we noticed that PL3 was unique in carrying the *fec* gene cluster on the pKPN plasmid compared with the other PL strains. Consequently, acquisition of the *fec* genes was considered in analysis of genetic traits suspected to increase FDC MIC of PL3. Interest in the FEC transport system was also corroborated by genomic analysis of other *K. pneumoniae* clinical isolates in our collection, given that isolates showing higher FDC MICs also carried the *fec* gene cluster.

With regard to the simplified *E. coli* K-12 laboratory strain model, we speculate that iron imported via the plasmid-encoded FEC system is sufficient to downregulate the expression of Fiu, CirA, FepA and FhuA iron transporters that also mediate FDC import (*37*,*41*,*42*). High intracellular iron levels activate the ferric uptake regulator protein, causing general repression of TonB-dependent transporters (*43*).

A recent study reported correlation of FDC resistance with a *fec* gene cluster, originating from *E. coli*, located on an IncC plasmid in VIM-1–producing Enterobacterales (*44*). Our findings extend that observation, demonstrating the effect of the widely diffused plasmid-mediated *fec* gene cluster in globally spread *K. pneumoniae* KPC-carbapenemase producers. In our model, the combination of 2 plasmids (e.g., pKPN and pKpQIL) resulted in reduced susceptibility to FDC. pKpQIL is one of the most diffused plasmids carrying *bla*_KPC_ gene variants. pKPN is a plasmid that seems to be restricted to *K. pneumoniae* and was initially recognized as a vehicle of the *fec* gene cluster in ST307 (*30*). We show that the *fec* gene cluster is present in many *K. pneumoniae* strains, including those isolated before introduction of FDC in clinical therapy.

Our most relevant evidence is that FDC resistance can be driven by genetic determinants located on plasmids, the success and spread of which occurred independently from the introduction of FDC for therapy. In the future, FDC may act as a positive selector for plasmids carrying the *fec* genes, for which prevalence can be expected to increase.

The results obtained in the *E. coli* experimental model should not allow extrapolation or prediction about the clinical efficacy of FDC in treating infections sustained by *fec*-positive *K. pneumoniae*. However, our study sets the background for future clinical studies aimed at testing the therapeutic efficacy of FDC in infections caused by *K. pneumoniae* carrying different combinations of plasmid-encoded carbapenemases and iron-uptake systems.

During infection, bacteria are faced with the low iron availability imposed by the iron-withholding response of the host (*45*) and must therefore express their iron uptake systems for successful tissue invasion and systemic spread (*46*). Citrate concentrations in biological fluids (≈100 μM in blood) (*47*) are high enough to activate the *fec* gene cluster; accordingly, selective expression of the FEC system has been documented to contribute to in vivo fitness of *E. coli* in human and animal infection (*48*–*50*). Moreover, the introduction of the CZA combination has contributed to selecting *K. pneumoniae* clinical strains that produce CZA-resistant KPC variants, such as KPC-31, characterized by very efficient cephalosporinase activity on the cephalosporin moiety of FDC (*38*).

Our novel finding is that the combination of a CZA-resistant KPC variant with the FEC system in *K. pneumoniae* may substantially increase the FDC MICs. Thus, the Trojan horse approach is a smart and effective strategy for delivering an antimicrobial drug to its target(s), but *K. pneumoniae* is equipped with plasmids that could help escape that trap.

Appendix 1 Additional information for study of cefiderocol resistance conferred by plasmid-located ferric citrate transport system in *Klebsiella pneumoniae* carbapenemase**–**producing *K. pneumoniae*.

Appendix 2 Presence and absence of *fec* gene cluster, *bla*_KPC_, *bla*_NDM_, *bla*_VIM_, and FIB(K) replicon in *Klebsiella pneumoniae* isolates downloaded from the Pasteur Institute BIGSdb database (https://bigsdb.pasteur.fr).

Appendix 3 Total positives-negatives for *fec* gene cluster for *Klebsiella pneumoniae* isolates downloaded from the Pasteur Institute BIGSdb database (https://bigsdb.pasteur.fr).

Appendix 4 Single-nucleotide polymorphism differences identified in PL1, PL2, PL3, PL4 strains analyzed in this study.

Appendix 5 BLAST results of *fec* gene cluster search in all *Klebsiella pneumoniae* isolates downloaded from the Pasteur Institute BIGSdb database (https://bigsdb.pasteur.fr).
