# Supplementary material for: Cefiderocol Resistance Conferred by Plasmid-Located Ferric Citrate Transport System in KPC-Producing Klebsiella pneumoniae
Source: Emerg Infect Dis. 2025 Jan;31(1):123–4. doi: 10.3201/eid3101.241426 (PMC11682805; doi:10.3201/eid3101.241426)
Supplement: Appendix 1 — Additional information for study of cefiderocol resistance conferred by plasmid-located ferric citrate transport system in Klebsiella pneumoniae carbapenemase–producing K. pneumoniae. [file 24-1426-Techapp-s1.pdf]

Article DOI: https://doi.org/10.3201/eid3101.241426

EID cannot ensure accessibility for supplementary materials supplied by authors. Readers who have difficulty accessing supplementary content should contact the authors for assistance.

Cefiderocol Resistance Conferred by Plasmid-Located Ferric Citrate Transport System in *Klebsiella pneumoniae* Carbapenemase–Producing *K. pneumoniae*

Appendix 1

Appendix 1 Table 1. Susceptibility to Cefiderocol of KPC-producing *Klebsiella pneumoniae* and bla_{KPC}-variants cloned in pTOPO vector in Top-10 *Escherichia coli*

Strain	MLST	Carbapenemase	FDC MIC mg/L	FecA	Ybt	OmpK35; OmpK36	DOI Reference	Acc. Number NCBI
K. pneumoniae								
PL3	ST512	KPC-31, KPC-31	4.0	Pos	Neg	ΔOmpK35; OmpK36GD	This study	PRJNA1139702
PL1	ST512	KPC-3, KPC-3	0.5	Neg	Neg	ΔOmpK35; OmpK36GD	This study	PRJNA1139702
PL2	ST512	KPC-3, KPC-31	2.0	Neg	Neg	ΔOmpK35; OmpK36GD	This study	PRJNA1139702
PL4	ST512	KPC-3, KPC-31	2.0	Neg	Neg	ΔOmpK35; OmpK36GD	This study	JBFRVZ000000000
1	ST512	KPC-3	2.0	Pos	Neg	ΔOmpK35; OmpK36GD	10.1128/AAC.00574-21	GCA_022341665.1
Co2	ST101	KPC-3	2.0	Pos	ybt 9; ICEKp3	ΔOmpK35; OmpK36DT	10.1016/j.ijantimicag.2020.106245	GCA_016807535.1
3B	ST512	KPC-3	2.0	Pos	Neg	ΔOmpK35; OmpK36GD	10.1128/AAC.00574-21	GCA_022341765.1
3	ST307	KPC-3	0.25	Neg	ybt 10; ICEKp4	ΔOmpK35; OmpK36 wt	10.1128/aac.00368-23	GCA_022341645.1
6	ST512	KPC-31	4.0	Pos	Neg	ΔOmpK35; OmpK36GD	10.1128/AAC.00574-21	GCA_022341565.1
1186W	ST512	KPC-31	2.0	Neg	ybt 9; ICEKp3	ΔOmpK35; OmpK36GD	10.3201/eid2911.230921	PRJNA992043
1186T	ST512	KPC-31	2.0	Neg	ybt 9; ICEKp3	ΔOmpK35; OmpK36GD	10.3201/eid2911.230921	PRJNA992043
1021	ST37	KPC-31	2.0	Neg	ybt 9; ICEKp3	wt	10.1099/mgen.0.000931	GCA_025118435.1
1016	ST37	KPC-31	2.0	Neg	ybt 9; ICEKp3	wt	10.1099/mgen.0.000931	SAMN29397000
21	ST307	KPC-31	1.0	Neg	ybt 10; ICEKp4	wt	10.1128/AAC.00574-21	GCA_022341545.1
1001	ST307	KPC-31	2.0	Neg	ybt 10; ICEKp4	OmpK35 wt; ΔOmpK36	10.1128/aac.00368-23	GCA_026127975.1
0213	ST307	KPC-31	1.0	Neg	ybt 10; ICEKp4	wt	10.1128/aac.00368-23	GCA_026128035.1
27B	ST307	KPC-31	1.0	Neg	ybt 10; ICEKp4	wt	10.1128/aac.00368-23	GCA_022341525.1
0323	ST307	KPC-31, KPC-3, KPC-3	2.0	Neg	ybt 10; ICEKp4	wt	10.1128/aac.00368-23	GCA_026127985.1
0296	ST512	VIM, KPC-3 (ΔCirA)	32.0	Pos	ybt 9; ICEKp3	ΔOmpK35; OmpK36mut	10.3201/eid2911.230921	PRJNA992043
6379	ST512	VIM, KPC-3	8.0	Neg	ybt 9; ICEKp3	ΔOmpK35; OmpK36GD	10.3201/eid2911.230921	PRJNA992043

Strain	MLST	Carbapenemase	FDC MIC mg/L	FecA	Ybt	OmpK35; OmpK36	DOI Reference	Acc. Number NCBI
2B	ST512	KPC-29	2.0	Pos	Neg	ΔOmpK35; OmpK36GD	10.1128/AAC.00574-21	GCA_022341715.1
1B	ST512	KPC-49	2.0	Pos	Neg	ΔOmpK35; OmpK36GD	10.1128/AAC.00574-21	GCA_022341825.1
4	ST512	KPC-66	2.0	Pos	Neg	ΔOmpK35; OmpK36GD	10.1128/AAC.00574-21	GCA_022341745.1
7	ST101	KPC-68	4.0	Pos	ybt 9; ICEKp3	ΔOmpK35; OmpK36DT	10.1128/AAC.00574-21	GCA_022341785.1
20B	ST101	KPC-68	4.0	Pos	ybt 9; ICEKp3	ΔOmpK35; OmpK36DT	10.1128/AAC.00574-21	GCA_022341705.1
40B	ST111	KPC-69	2.0	Pos	ybt 7; ICEKp7	wt	10.1128/AAC.00574-21	GCA_022341485.1
42B	ST512	KPC-70	4.0	Pos	Neg	ΔOmpK35; OmpK36GD	10.1128/AAC.00574-21	GCA_022341915.1
9	ST101	KPC-39	1.0	Neg	ybt 9; ICEKp3	ΔOmpK35; OmpK36DT	10.1128/AAC.00574-21	GCA_022341495.1
26B	ST512	KPC-66	2.0	Neg	Neg	ΔOmpK35; OmpK36 wt	10.1128/AAC.00574-21	GCA_022341685.1
17B	ST512	KPC-67	1.0	Neg	Neg	ΔOmpK35; OmpK36GD	10.1128/AAC.00574-21	GCA_022341815.1
2	ST512	KPC-67	2.0	Neg	Neg	ΔOmpK35; OmpK36GD	10.1128/AAC.00574-21	GCA_022341885.1
10	ST512	KPC-67	2.0	Neg	Neg	ΔOmpK35; OmpK36GD	10.1128/AAC.00574-21	GCA_022341865.1
13	ST512	KPC-67	2.0	Neg	Neg	ΔOmpK35; OmpK36GD	10.1128/AAC.00574-21	GCA_022341805.1
6099	ST512	KPC-154	1.0	Neg	ybt 9; ICEKp3	ΔOmpK35; OmpK36GD	10.3201/eid2911.230921	PRJNA992043
1020	ST37	KPC-110	2.0	Neg	ybt 9; ICEKp3	wt	10.1099/mgen.0.000931	GCA_025118455.1
Top-10 *E. coli*								
Top-KanR	-	-	0.064	-	-	-	10.1128/AAC.00574-21	-
KPC-3-TOPO	-	-	0.125	-	-	-	10.1128/AAC.00574-21	-
KPC-29-TOPO	-	-	0.125	-	-	-	10.1128/AAC.00574-21	-
KPC-154-TOPO	-	-	0.125	-	-	-	10.3201/eid2911.230921	-
KPC-67-TOPO	-	-	0.25	-	-	-	10.1128/AAC.00574-21	-
KPC-39-TOPO	-	-	0.5	-	-	-	This study	-
KPC-66-TOPO	-	-	0.5	-	-	-	10.1128/AAC.00574-21	-
KPC-69-TOPO	-	-	0.5	-	-	-	10.1128/AAC.00574-21	-
KPC-110-TOPO	-	-	0.5	-	-	-	10.1099/mgen.0.000931	-
KPC-31-TOPO	-	-	1.0	-	-	-	10.1128/AAC.00574-21	-
KPC-68-TOPO	-	-	1.0	-	-	-	10.1128/AAC.00574-21	-
KPC-70-TOPO	-	-	2.0	-	-	-	10.1128/AAC.00574-21	-

Appendix Table 2. List of primers used in this study and relative application

Primer name	Sequence	Application
cml_sma_F	CTCCTGATGATGCATGGTTACTCACCACTGCGATCCCC TCTGTATTAACGAAGCGCTAACC	R69c assembly
cml_sma_R	AATCAGGATATTCTTCTAATACCTGGAATGCTGTTTTCC CCCTGGTGTCCCTGTTGATACC	R69c assembly
R69_pmefec_F	AACCAGATTATTGATCTTCTCAACCAGCTCCGGGCGTT TTTCAGGCATGGTACATCCGCGG	R69c-FEC assembly and check
R69_pmefec_R	CGATATTACAAAAAGGGCAGCGCCCACATTACGTTTGA CTTCGCCAGACTACTTCACC	R69c-FEC assembly and check
CMPCR_F	TAGTCAATAAACCGGTAAACCAG	R69c check
CMPCR_R	CCTGGTGTCCCTGTTGATACC	R69c check
LM_F	GGATGAAAACTATCAGCATCTGAAG	R69c/R69cFEC conjugation check
LM2_R	GAACTCCGGCGAAAGACCTTC	R69c/R69cFEC conjugation check
KPC_PROM_F	GATCCAGGTGGGTCAGTATTACT	pKpQIL transformation and conjugation check
KPC-R	TTCAGAGCCTTACTGCCCGT	pKpQIL transformation and conjugation check
qRT_fiu_F	CGTTTTTTGCGGGTGAGAA	mRNA expression determination
qRT_fiu_R	CGGCACCACGCATATAAATG	mRNA expression determination
qRT_cirA_F	CGCGTGCCTACCTGGTTTAT	mRNA expression determination
qRT_cirA_R	CAGCCCCCTTTCACCGTTA	mRNA expression determination
qRT_fepA_F	TCGAAATATGGCGATGAAACC	mRNA expression determination
qRT_fepA_R	TTCCAGGTCAGCGCGTAGT	mRNA expression determination
qRT_fhuA_F	CTGGCGTCCGGATGATAAA	mRNA expression determination
qRT_fhuA_R	TCCGGCTCGTTCTGGAAGT	mRNA expression determination
crom_fecA_F	CGGGTATGCGTTTCGAACA	mRNA expression determination
crom_fecA_R	CTTCTTCGTGCGTGCCTG	mRNA expression determination
pcfecA_F	CGTCAATGGCATCCATGTTG	mRNA expression determination
pcfecA_R	TACGGTCAGCCGCAGCTT	mRNA expression determination

Appendix Table 3. Cefiderocol Minimal Inhibitory Concentrations values with respective average and standard deviation values

			FDC MIC mg/L, [NH+4]5 [Fe(C6H4O7)2]5-														
			0,0 μM					0,5 μM					5,0 μM				
	Strain	KPC	M1	M2	M3	Average	Standard Deviation	M1	M2	M3	Average	Standard Deviation	M1	M2	M3	Average	Standard Deviation
Plasmid	DH5alpha		0,064	0,064	0,25	0,13	0,11										
R69c			0,125	0,25	0,125	0,17	0,07										
	FEC		0,25	0,25	0,25	0,25	0										
pKpQIL	3	KPC-3	0,25	0,125	0,25	0,21	0,07										
	PL1	KPC-3; KPC-3	0,125	0,25	0,25	0,21	0,07										
	42B	KPC-70	0,5	0,5	0,5	0,5	0										
	PL4	KPC-3; KPC-31	0,5	1	1	0,83	0,29										
	1021	KPC-31	1	1	1	1	0										
	PL3	KPC-31; KPC-31	1	1	1	1	0										

			FDC MIC mg/L, [NH+4]5 [Fe(C6H4O7)2]5-														
			0,0 µM					0,5 µM					5,0 µM				
	Strain	KPC	M1	M2	M3	Average	Standard Deviation	M1	M2	M3	Average	Standard Deviation	M1	M2	M3	Average	Standard Deviation
Plasmid	DH5alpha		0,064	0,064	0,25	0,13	0,11										
R69c - pKpQIL	3	KPC-3	0,25	0,25	0,25	0,25	0	0,5	0,5	0,5	0,5	0	1	1	1	1	0
	PL1	KPC-3; KPC-3	0,5	0,5	0,5	0,5	0	0,5	0,5	0,5	0,5	0	1	1	1	1	0
	42B	KPC-70	0,5	0,5	0,5	0,5	0	1	1	1	1	0	1	1	1	1	0
	PL4	KPC-3; KPC-31	0,5	1	1	0,83	0,29	1	1	1	1	0	1	1	1	1	0
	1021	KPC-31	1	0,5	1	0,83	0,29	1	1	1	1	0	2	2	2	2	0
	PL3	KPC-31; KPC-31	1	1	1	1	0	2	1	2	1,67	0,58	4	2	4	3,33	1,15
R69cFEC - pKpQIL	3	KPC-3	0,5	0,5	0,5	0,5	0	1	1	1	1	0	1	1	1	1	0
	PL1	KPC-3; KPC-3	1	1	1	1	0	1	1	1	1	0	2	2	2	2	0
	42B	KPC-70	4	2	2	2,67	1,15	2	2	2	2	0	4	4	4	4	0
	PL4	KPC-3; KPC-31	2	2	2	2	0	4	4	4	4	0	8	8	8	8	0
	1021	KPC-31	2	2	2	2	0	2	2	2	2	0	8	8	8	8	0
	PL3	KPC-31; KPC-31	4	4	4	4	0	8	8	8	8	0	16	16	16	16	0

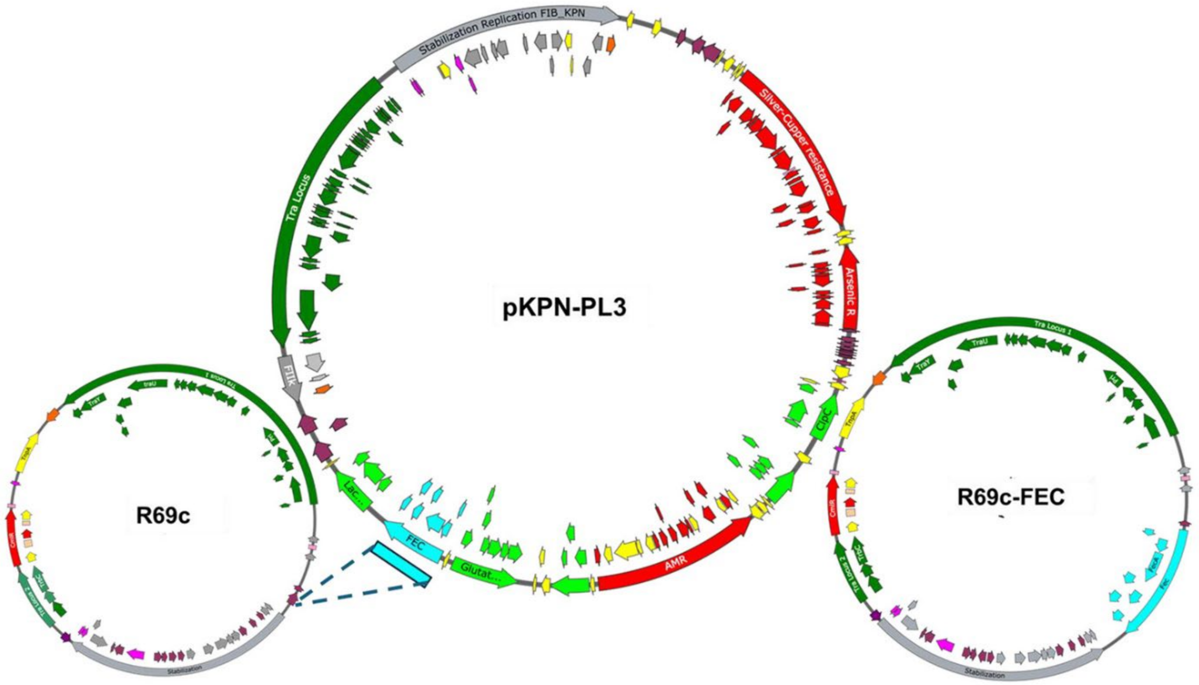

Appendix Figure 1. Schematic representation of R69c-FEC construction. Major features of plasmids in circular maps indicated by coloured arrows: green Tra Locus loci (R69c includes *excA, traY, traX, traW, traU, traR, traQ, traP, traO, traN, traM, traL, pri, traK, traJ, traI, traH, mobA, mobB, tir*, *trbA, trbB* and *trbC*); grey stabilization genes (R69c carries *ssB, klcA, korC, radC, nuc, parB, parA, resD, relB, mucB, mucA)*; pink, *pemK* (toxin), *pemI* (antitoxin) genes; red, resistance gene region; orange, replicons; pale blue, *fec* gene cluster; light green, metabolic or virulence cluster in pKPN-PL3.

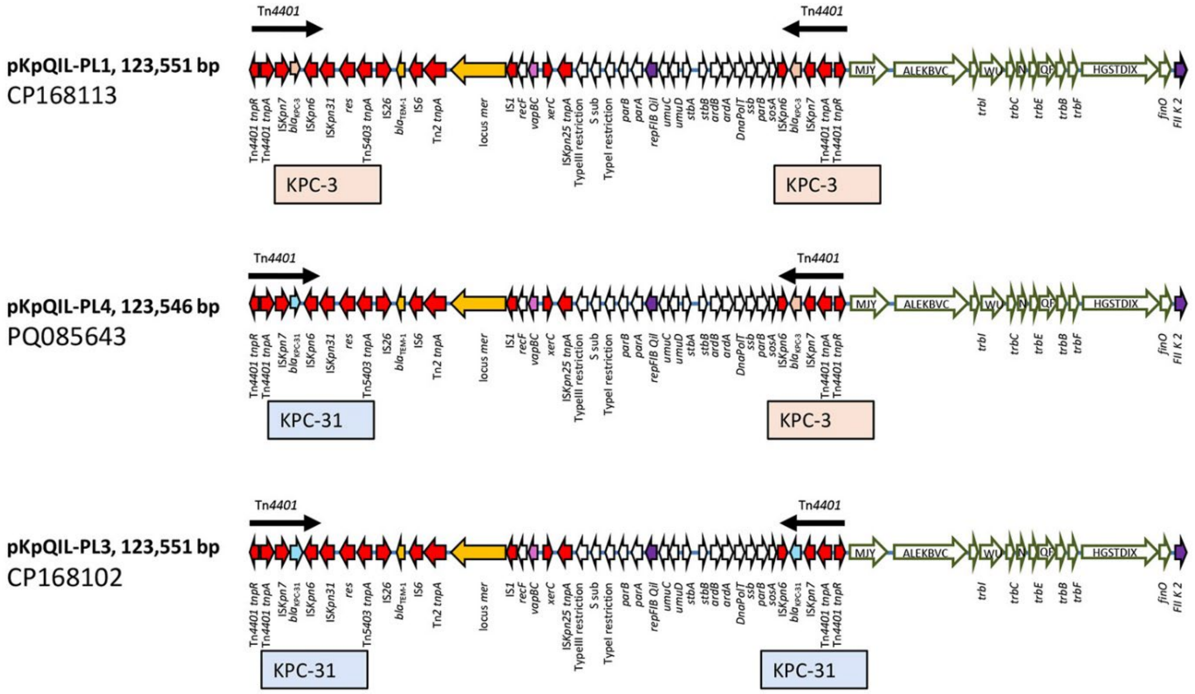

Appendix Figure 2. Maps of the pKpQIL plasmids**.** Major structural features of pKpQIL-PL1, pKpQIL-PL4, and pKpQIL-PL3 identified in ST512 *K. pneumoniae* isolates PL1, PL4 and PL3, respectively. Predicted coding sequences are indicated by coloured arrows oriented in the direction of transcription of each gene. Resistance genes, yellow, except the bla_{KPC-3} arrows are pink and bla_{KPC-31} arrows are in pale blue; transposon-related genes and insertion sequences, red; replicons, violet; other genes, white. The green bordered arrows indicate *tra* locus and the associated *trb* and *finO* genes. The position of the double bla_{KPC} copies is highlighted by black arrows indicating the position of the Tn*4401* transposons above the maps, and coloured boxes below the maps: bla_{KPC-3}, pink; bla_{KPC-31}, pale blue. The sizes of the arrows are not to scale.

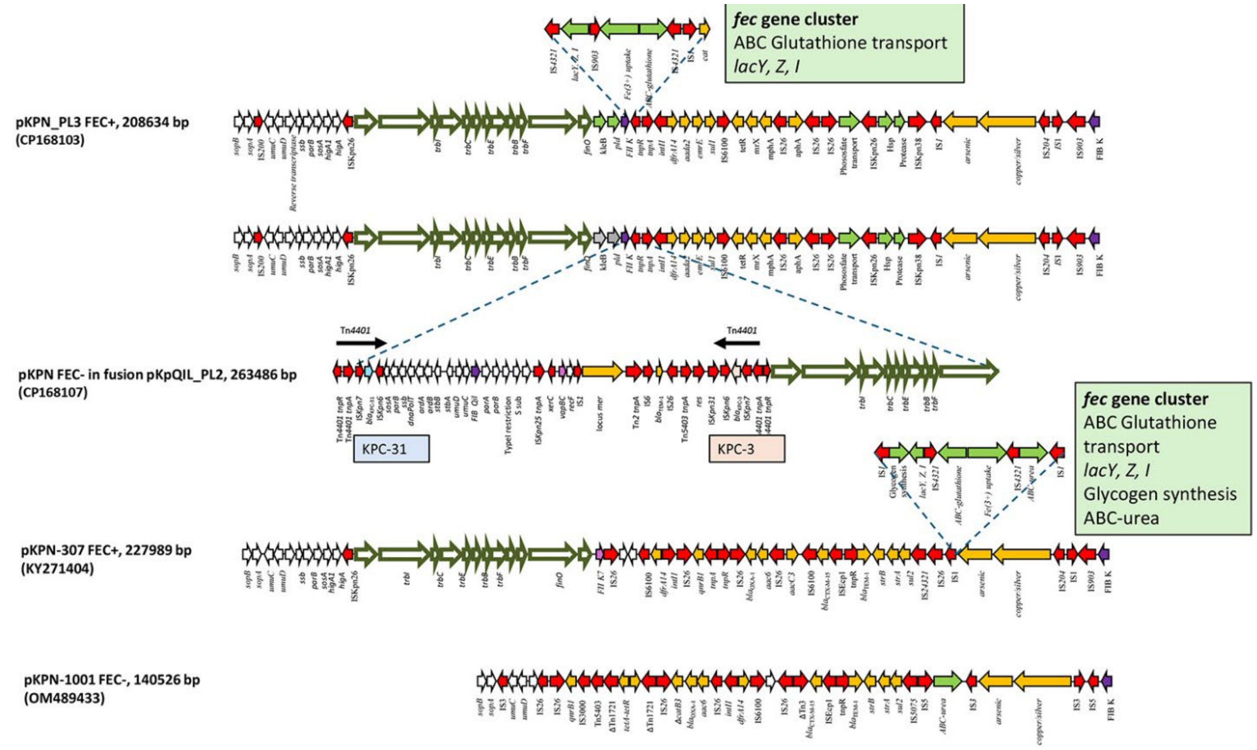

Appendix Figure 3. Figure S3. Maps of pKPN plasmids. Major structural features of pKPN-PL3, pKPN-PL2 identified in PL3 and PL2 *K. pneumoniae* isolates, respectively. pKPN-PL2 is fused with pKpQIL-PL2 plasmid (map and integration site are indicated below the pKPN-PL2 map by dot lines). Maps of the two reference pKPN-307 and pKPN-1001 plasmids representing the largest and smaller pKPN plasmid identified in our previous studies in ST307 and ST512 *K. pneumoniae* isolates, respectively are also included for comparison. Predicted coding sequences are indicated by coloured arrows oriented in the direction of transcription of each respective gene: resistance genes, yellow, except the $bla_{\text{KPC-3}}$ arrows are pink and $bla_{\text{KPC-31}}$ arrows are in pale blue; transposon-related genes and insertion sequences, red; replicons, violet; klebicin cluster, grey; other genes, white. The green bordered arrows indicate *tra* locus and the associated *trb* and *finO* genes. The position of the double bla_{KPC} copies is highlighted by coloured boxes below the maps: $bla_{\text{KPC-3}}$, pink; $bla_{\text{KPC-31}}$, pale blue. Virulence clusters acquired by pKPN and their integration sites are reported as separated units connected by dot lines and their content is highlighted by green boxes above the maps. The sizes of the arrows are not to scale.

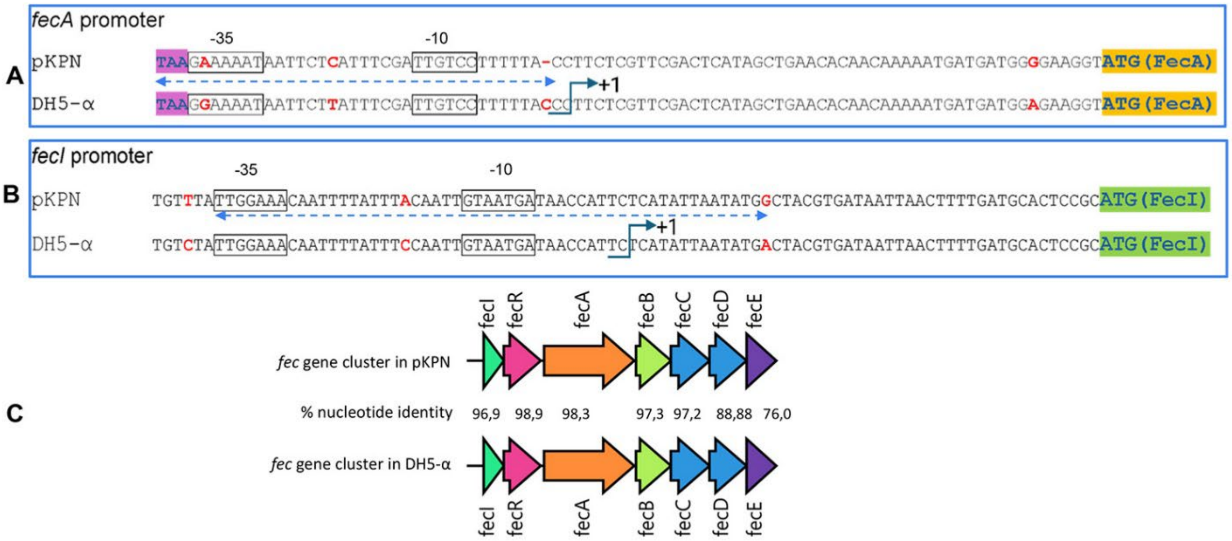

Appendix Figure 4. Comparion of the *fec* gene cluster in the pKPN plasmid and the chromosomal *fec* gene cluster in *Escherichia coli* DH5-α Experimentally determined Fur binding sites are defined by dotted double arrows. The -10 and -35 hexamers are boxed. The +1 arrow indicates the experimentally determined transcription initiation site in *E. coli* (*1*,*2*). A) *fecA* promoter: the stop codon of *fecR* is highlighted in magenta, the ATG start codon of *fecA* is in orange; B) *fecI* promoter: the start codon of *fecI* is highlighted in green. C) Physical map of the *fec* genes in the cluster and percentage of nucleotide identity between each gene of the cluster: *fecI* and *fecR* are the regulatory genes, and *fecABCDE* is the transport operon.

References

1. Enz S, Mahren S, Menzel C, Braun V. Analysis of the ferric citrate transport gene promoter of *Escherichia coli.* J Bacteriol. 2003;185:2387–91. https://doi.org/10.1128/JB.185.7.2387-2391.2003

2. Angerer A, Braun V. Iron regulates transcription of the *Escherichia coli* ferric citrate transport genes directly and through the transcription initiation proteins. Arch Microbiol. 1998;169:483–90. https://doi.org/10.1007/s002030050600
